# Catching Up to Increased Complexity in Breast Cancer Molecular Testing

**DOI:** 10.1016/j.jmoldx.2025.03.007

**Published:** 2025-04-23

**Authors:** Gregory R. Bean, Chieh-Yu Lin, Melissa Krystel-Whittemore, Lulu Sun

**Affiliations:** ∗ Breast Cancer Webinar Series Content Committee, A Working Group of the Training and Education Committee, Association for Molecular Pathology, Rockville, Maryland; † Department of Pathology, Stanford University, Stanford, California; ‡ Department of Pathology and Immunology, Washington University in St. Louis, St. Louis, Missouri; § Medical Diagnostics, US Medical Affairs, Oncology, AstraZeneca, Wilmington, Delaware

In recent years, continued research efforts have yielded new biomarkers for invasive breast cancer (IBC), as well as a deeper understanding of its heritability. The Association for Molecular Pathology issued a four-part webinar series—Breast Cancer Molecular Testing: Catching Up to Increased Complexity (*https://www.amp.org/breastcancer*, last accessed February 18, 2025)–to provide pathologists, geneticists, oncologists, genetic counselors, laboratory professionals, and other members of the patient care team with a comprehensive overview of this rapidly evolving field. This Practice Advance paper summarizes the key points from this series.

## Update on Breast Cancer Biomarkers

Biomarkers can be diagnostic, prognostic, and/or predictive. Estrogen receptor (ER) is an example of a biomarker that can act as all of the above in IBC. Progesterone receptor (PR) is primarily a prognostic marker. Depending on the biomarker and what it is trying to prognosticate or predict, the thresholds for positivity may vary. For example, ER positivity of ≥1% in tumor cells can predict benefit from adjuvant endocrine therapy, but ≥10% may be a more suitable cutoff in terms of prognosis and clinical trial enrollment.

Biomarkers may also be differentially relevant at different stages, time points, and subgroups of IBC treatment. In the post-neoadjuvant setting, pathologic complete response is a major prognostic biomarker. With residual cancer, patients can become eligible for abemaciclib (a CDK4/6 inhibitor) if they have high-risk, ER^+^ disease, or for the antibody–drug conjugate trastuzumab-emtansine (TDM1) if the tumor is HER2^+^. If the tumor is ER/PR/HER2 triple-negative (TNBC), the immune checkpoint inhibitor pembrolizumab might be continued, or the chemotherapeutic agent capecitabine could be added.

In the metastatic setting, ER and HER2 status continue to determine the overall therapy. ER^+^ HER2^−^ IBCs with certain somatic mutations may be targeted by specific therapies (further discussed below). IBC patients with a HER2 immunohistochemistry (IHC) score of 1+ or 2+ (with negative *in situ* hybridization) are now candidates for the antibody–drug conjugate trastuzumab deruxtecan. For TNBC, pembrolizumab may be administered along with chemotherapy if the PD-L1 22C3 combined positive score is ≥10. Finally, some treatments do not require a biomarker, for example, sacituzumab govitecan targeting TROP2.

## Somatic Tumor Testing

The current treatment landscape of IBC has been impacted by new predictive biomarkers with accompanying US Food and Drug Administration (FDA)-approved targeted therapies that can be evaluated using somatic molecular testing.

*PIK3CA* mutations (most commonly missense mutations) are seen in approximately 35% to 40% of ER^+^ HER2^−^ IBC, approximately 22% of HER2^+^ BC, and approximately 18% of TNBC. In 2019, the FDA approved alpelisib (PIK3CA p110α inhibitor) for *PIK3CA*-mutated advanced HR^+^ HER2^−^ IBC. Additionally, in 2023, capivasertib (AKT1 inhibitor) was FDA-approved for the treatment of HR^+^ HER2^−^ IBC with *PIK3CA, AKT1*, or *PTEN* alterations. Testing for these alterations is typically performed using next-generation sequencing or PCR testing.

*ESR1* alterations are commonly gain-of-function missense mutations in the ligand-binding domains (codons 310 to 547), which lead to constitutive ER activity and acquired resistance to aromatase inhibitors. *ESR1* mutations are rarely seen in primary breast tumors but are identified in approximately 30% of metastatic ER^+^ BC after receiving aromatase inhibitors. Elacestrant was FDA-approved in 2023 for the treatment of *ESR1-*mutated ER^+^ HER2-advanced IBC after at least one line of endocrine therapy.

Although HER2 amplification is much more common in IBC, *ERBB2* missense mutations within the kinase domain can also be observed, leading to signal activation without HER2 amplifications. *ERBB2*-activating mutations are observed in approximately 3% to 4% of IBC cases. These tumors are less sensitive to trastuzumab and lapatinib, but are sensitive to neratinib.

*BRCA1/2* mutations are seen in approximately 5% to 10% of all IBC and 75% to 80% of hereditary IBC cases. In 2018, the FDA approved olaparib (PARP inhibitor) for patients with germline *BRCA*-mutated IBC. The National Comprehensive Cancer Network has also added a 2B recommendation for PARP inhibitor treatment in patients with somatic *BRCA* mutations.

Tumor-type agnostic markers have also impacted treatment options for IBC. Tumors with increased PD-L1 expression (evaluated by IHC), mismatch repair deficiency (IHC, PCR, or next-generation sequencing), and high tumor mutational burden (next-generation sequencing) may be eligible for immune checkpoint inhibitor therapy. Additionally, although rare, *NTRK* and *RET* fusions can be observed in IBC, with larotrectinib and entrectinib available for the former and selpercatinib for the latter as treatment options.

Somatic tumor testing for the above genomic alterations may be performed on tissue, or on circulating tumor DNA (ctDNA) obtained from blood. ctDNA testing approaches are minimally invasive and allow for repeat, longitudinal testing, and may be particularly useful in monitoring for *ESR1* mutations. The sensitivities of ctDNA tests vary and a negative result does not always rule out the presence of a somatic variant.

## Multi-Gene Assays

Risk assessment is particularly important for patients with luminal intrinsic type IBC, a heterogeneous group of tumors with strong ER expression. Compared to Luminal A IBC, Luminal B IBC tends to have worse outcomes and warrants more aggressive treatment. However, IHC alone is not reliable for discerning subtypes. Multi-gene expression assays (MGA) have thus been developed to determine which patients have good prognosis and can forgo aggressive treatment.

Oncotype DX is used to assess the benefit of adjuvant chemotherapy for ER^+^ breast cancer with 0 to 3 positive nodes. It includes 16 cancer-related genes with 5 housekeeping genes and generates a recurrence score (RS). For node-negative patients [Trial Assigning IndividuaLized Options for Treatment (Rx) (TAILORx trial)], tumors with RS ≤ 10 can be treated with adjuvant hormone therapy alone, whereas patients with RS ≥ 26 benefit from adjuvant chemotherapy. Within the group of patients with RS 11 to 25, premenopausal patients might benefit from adjuvant chemotherapy. For node-positive disease [one to three positive nodes; A Clinical Trial RX for Positive Node, Endocrine Responsive Breast Cancer (RxPONDER trial)], patients with RS ≥ 26 may benefit from adjuvant chemotherapy. For premenopausal patients with RS < 26, chemotherapy provides some clinical benefit, which might be due to ovarian suppression.

MammaPrint is a 70-gene MGA. The MINDACT (Microarray In Node negative and 1 to 3 positive lymph node Disease may Avoid ChemoTherapy) trial showed that MammaPrint can help identify a subset of clinically high-risk patients with excellent prognosis who can forego adjuvant chemotherapy. There is no benefit in performing MammaPrint for the clinically low-risk patient group. MammaPrint gives a binary low-risk or high-risk result, which are further subdivided into four groups (Ultra-low, Low, High 1, and High 2), providing additional risk stratification for de-escalation of adjuvant endocrine therapy. BluePrint, a related 80-gene MGA, provides an estimation of the underlying biology/intrinsic type.

The Breast Cancer Index (BCI) assay evaluates the ratio of *HOBX13*/*IL17BR* and five proliferation genes. It is predictive of late distant recurrence (5 to 10 years) for ER^+^ node-negative breast cancer. It can thus aid in deciding endocrine therapy duration.

For surgical pathologists, when choosing blocks for MGA testing, it is ideal to pick a block with predominant invasive carcinoma (ie, less *in situ* component or other proliferative lesions), highest grade of tumor, and to avoid the biopsy cavity. When discrepancies between ER/PR/HER2 IHC and MGA results arise, IHC is still considered the gold standard, and a comment in the report is warranted.

## Germline Testing in IBC

Germline testing serves many purposes for IBC. The results may affect treatment decisions, including choice of surgical approach (eg, prophylactic contralateral mastectomy) and choice of adjuvant therapy (eg, PARP inhibitors). These findings are also significant for secondary prevention and screening for additional tumor types, such as ovarian cancer. Finally, germline testing results are important for family members to plan for risk reduction and cancer screening.

Guidelines differ in recommending which patients should undergo testing based on age and personal/family history. Expanding the criteria increases the number of tested patients and the sensitivity of detection; however, this escalates the resources required, including genetic counseling.

There are also concerns about which genes to include in breast cancer susceptibility assays. As sequencing technology has advanced, multi-gene panels beyond *BRCA1/2* have become more commonplace, with the inclusion of additional high-penetrance genes such as *PALB2*, *PTEN*, *TP53*, and genes that have lower breast cancer risk or moderate penetrance, including *CHEK2*, *ATM*, *RAD51C*, and *RAD51D*. However, there is little information regarding variants in these additional genes, resulting in many variants of uncertain significance with larger gene panels. Variants of uncertain significance are also more commonly found in patients of non-European ancestry due to fewer sequenced genomes/tumors in these populations. This lack of data/evidence may contribute to existing racial disparities, with higher breast cancer mortality rates in Black women than in White women in the United States.

Polygenic risk scores, which incorporate numerous susceptibility single nucleotide variants identified from genome-wide association studies, are an emerging promising tool for personalized risk assessment, including in non-White populations. As knowledge accumulates, consideration of ancestry will be important in the administration and interpretation of germline testing in breast cancer patients.

## Conclusions

Molecular testing of IBC is complex and involves numerous approaches, assays, and rationales. Significant hurdles to implementation of molecular testing still remain, including accessibility, affordability, and standardization. Tackling these issues will be essential to achieving the goals of precision medicine and health equity for breast cancer patients.

## Recommended Reading

Allison KH, Hammond MEH, Dowsett M, McKernin SE, Carey LA, Fitzgibbons PL, Hayes DF, Lakhani SR, Chavez-MacGregor M, Perlmutter J, Perou CM, Regan MM, Rimm DL, Symmans WF, Torlakovic EE, Varella L, Viale G, Weisberg TF, McShane LM, Wolff AC: Estrogen and progesterone receptor testing in breast cancer: American Society of Clinical Oncology/College of American Pathologists guideline update. Arch Pathol Lab Med 2020, 144:545–563

Bedrosian I, Somerfield MR, Achatz MI, Boughey JC, Curigliano G, Friedman S, Kohlmann WK, Kurian AW, Laronga C, Lynce F, Norquist BS, Plichta JK, Rodriguez P: Germline testing in patients with breast cancer: ASCO-Society of Surgical Oncology Guideline. J Clin Oncol 2024, 42:584–604

Burstein HJ, DeMichele A, Somerfield MR, Henry NL; Biomarker Testing and Endocrine and Targeted Therapy in Metastatic Breast Cancer Expert Panels: Testing for ESR1 mutations to guide therapy for hormone receptor-positive, human epidermal growth factor receptor 2-negative metastatic breast cancer: ASCO Guideline Rapid Recommendation Update. J Clin Oncol 2023, 41:3423–3425

Sun L, Wu A, Bean GR, Hagemann IS, Lin CY: Molecular testing in breast cancer: current status and future directions. J Mol Diagn 2021, 23:1422–1432

Wolff AC, Somerfield MR, Dowsett M, Hammond MEH, Hayes DF, McShane LM, Saphner TJ, Spears PA, Allison KH: Human epidermal growth factor receptor 2 testing in breast cancer: ASCO-College of American Pathologists guideline update. J Clin Oncol 2023, 41:3867–3872

## Disclaimer

The Association for Molecular Pathology (AMP) Practice Guidelines and Reports are developed to be of assistance to laboratory and other health care professionals by providing guidance and recommendations for particular areas of practice. The Guidelines or Reports should not be considered inclusive of all proper approaches or methods, or exclusive of others. The Guidelines or Reports cannot guarantee any specific outcome, nor do they establish a standard of care. The Guidelines or Reports are not intended to dictate the treatment of a particular patient. Treatment decisions must be made on the basis of the independent judgment of health care providers and each patient's individual circumstances. The AMP makes no warranty, express or implied, regarding the Guidelines or Reports and specifically excludes any warranties of merchantability and fitness for a particular use or purpose. The AMP shall not be liable for direct, indirect, special, incidental, or consequential damages related to the use of the information contained herein.

